# *Demodex* sp. as a Potential Cause of the Abandonment of Soft Contact Lenses by Their Existing Users

**DOI:** 10.1155/2015/259109

**Published:** 2015-07-21

**Authors:** Witold Tarkowski, Joanna Moneta-Wielgoś, Daniel Młocicki

**Affiliations:** ^1^Medical Centre KOL-MED SP ZOZ, Plac Dworcowy 6, 33-100 Tarnow, Poland; ^2^Department and Clinic of Ophthalmology, 1st Faculty of Medicine, Medical University of Warsaw, Ulica Lindleya 4, 02-005 Warsaw, Poland; ^3^Department of General Biology and Parasitology, Medical University of Warsaw, Ulica Chałubińskiego 5, 02-004 Warsaw, Poland; ^4^Institute of Parasitology, Polish Academy of Sciences, Ulica Twarda 51/55, 00-818 Warsaw, Poland

## Abstract

*Demodex* mites may be a potential etiological factor in the development of various eye and skin disorders. The aim of the study was to investigate the presence of *Demodex* in the hair follicles of eyelashes and their potential influence on abandoning soft contact lenses which had been previously well tolerated by their users. A group of 62 users of contact lenses (28 with emerging discomfort and 34 without discomfort) were examined. There is a need to check the existence of a relationship between *D. folliculorum* or/and *D. brevis* infestation and the emergence of intolerance to the presence of soft contact lenses. The removed lashes were examined under light microscopy, applying standard parasitological methods if demodicosis is suspected. A positive result was assumed if at least one adult stage, larva, protonymph/nymph, or egg of *D. folliculorum* and/or *D. brevis* was present. A positive correlation was observed between the presence of *Demodex* and intolerance to contact lenses by their existing users (*p* < 0.05), and *Demodex* sp. infections were observed in 92.86% of patients with intolerance to contact lenses. Our results provide further evidence for the pathogenic role played by the mites in the development of eye diseases.

## 1. Introduction

The first contact lenses were constructed from glass. In 1936, the American company Rohm and Haas introduced transparent plastic PMMA (Polymethyl methacrylate) lenses. In 1955, Wichterle and Lim, two chemists from Prague, invented PHEMA (hydroxyethyl methacrylate), and this material was used to create the first “soft” contact lens in 1961, thus making the mass production of more comfortable and cheaper contact lenses possible. The patent to develop these soft contact lenses commercially was subsequently acquired by Bausch and Lomb in 1965.

PHEMA lenses can be worn for long periods of time during the day and are better tolerated by the wearer. However, they can absorb microorganisms as well as secretions from the eye [1].

Over the past 40 years, huge improvements have been made in soft contact lens design. Frequent replacement lenses were commercialised in 1987, and in 1994, the first one-day lenses were produced, representing a milestone in the history of contactology [2]. In the late 1990s, silicone-hydrogel lenses with high oxygen permeability, that is, a high Dk coefficient, were introduced onto the market. This Dk value is elevated in hydrogel lenses due to the greater volume of water present, water being an efficient transporter of oxygen. However, the high Dk value of silicone-hydrogel lenses is associated with the presence of hydrophobic monomers such as silicone.

The use of new materials can eliminate many of the complications associated with oxygen deficiency in the cornea. This would allow lenses to be used by a greater number of people, including those who have had early problems with contact lens tolerance [3]. There has been a steady increase in individuals with refraction defects requiring correction, and some of these are users of contact lenses. It is estimated that, in the United States, various vision defect corrections are applied by more than 162 million people, or nearly 60% of the population, of whom about 22% wear contact lenses [4]. In contemporary practice, more attention is spent on caring for patients wearing contact lenses than to initially matching the lenses to the patient [5].

The use of bifocals and progressive lenses allows presbyopia to be corrected. Cosmetic lenses, which allow eye colour to be emphasized or changed, are gaining popularity as “dressing lenses” in treating superficial damage to the corneal epithelium occurring* inter alia* in spring, or inflammation of the giant-papilla conjunctiva, by protecting the cornea against mechanical damage caused by the papilla rubbing against the epithelium. New generation lenses which ensure high oxygen permeability also ensure the integrity of the eye surface in such diseases as filament keratitis, ocular cicatricial pemphigoid multiform erythema, chemical after-burn conditions, neurotrophic keratopathy, or abnormal lash growth [6].

An understanding of the possible causes of intolerance to contact lenses may therefore be of both medical and economic importance. Previous advances in contact lens design have made the lenses more tolerable. For example, the use of hydrogel-silicone materials with high oxygen permeability has eliminated the physiological changes experienced by users of lenses with low Dk. The morning corneal edema experienced in the users of new generation lenses is similar to the physiological edema observed with no lens present, and its occurrence is much lower than that seen in other frequent change soft lenses currently available in the market [2, 7–9]. The striations (striae), microcysts, and polymegathism usually associated with contact lenses are rarely observed, if at all, in users of hydrogel-silicone lenses with high gas permeability [7]. The lenses also reduce limbus congestion and eliminate the ingrowth of blood vessels in the limbus, leaving only the “ghosts of blood,” as experienced by users of earlier lenses with extended wear and low Dk values [7].

It is estimated that nearly half of contact lens users experience dry eye symptoms. Contact lens wearers who are not fully satisfied and feel even a little discomfort associated with their use often decide to shorten their use or forego them altogether [10]. Discomfort, particularly xerophthalmia, is the primary reason given for dissatisfaction with wearing soft contact lenses and accounts for about 50% of abandonments [11–14]. An understanding of the reasons for doing so could have a crucial impact on reducing this high incidence rate.

**Figure 1:**
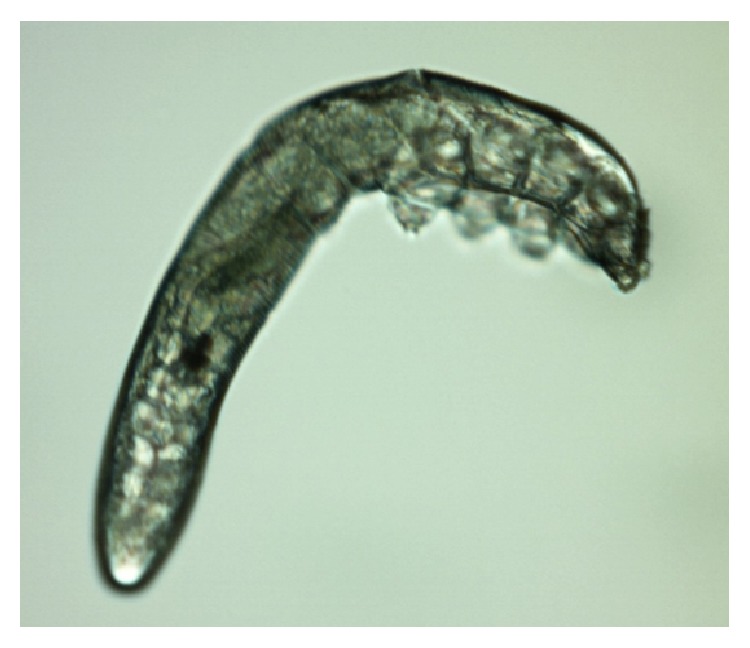
*D. folliculorum,* adult stage.

**Figure 2:**
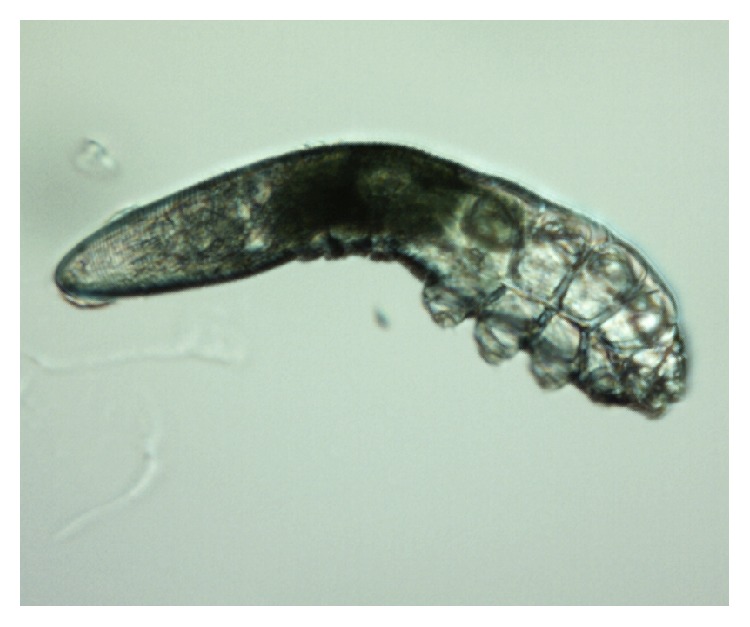
*D. brevis,* adult stage.

**Figure 3:**
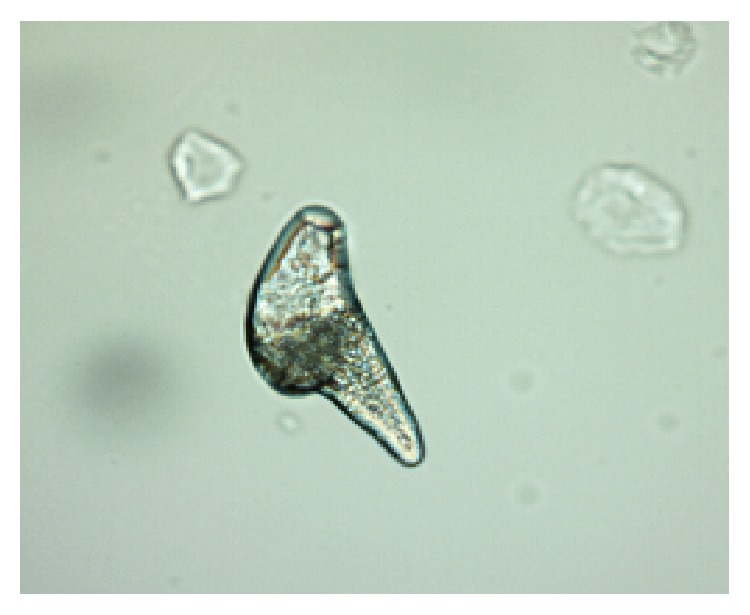
Egg of* D. folliculorum*.

**Figure 4 fig4:**
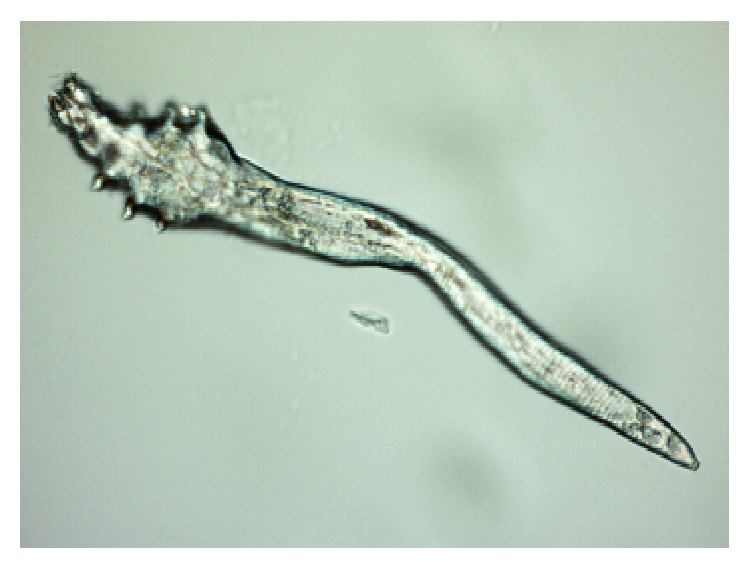
Larva of* D. folliculorum*.

**Figure 5 fig5:**
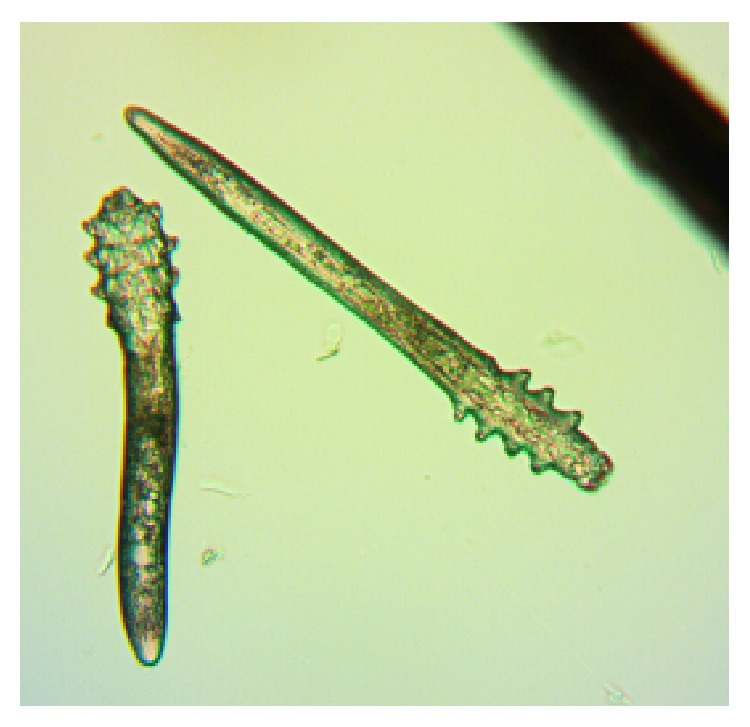
Protonymph and adult stage of* D. folliculorum*.

**Figure 6:**
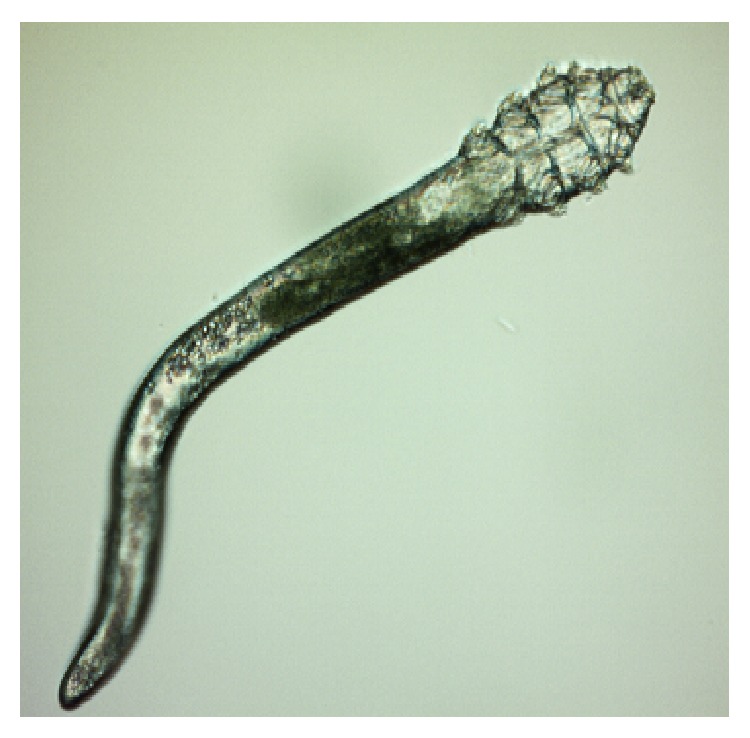
Nymph of* D. folliculorum*.

*Demodex* are cosmopolitan mites; so, their existence is recorded in many countries in all continents apart from Antarctica [15, 16]. Currently, more than 100 different species of the* Demodex* genus are recognised. They are highly specialized organisms which are obligatorily associated with their hosts, and different species of* Demodex* may be present on various parts of the skin of one host at any one time [17]. Two species of* Demodex* can exist on humans [18, 19]:* Demodex folliculorum* (Figures 1, 3 [Fig fig4] [Fig fig5]–6) and* Demodex brevis* (Figure 2).* D. folliculorum* is found in the orifice of hair follicles, where it creates clusters of small numbers of mites.* Demodex brevis* lives mainly alone in the depths of the sebaceous glands in the skin of face or in the Meibomian glands located within the eyelids [20]. It feeds on sebum and the gland cells, resulting in their destruction. It has been revealed that both species of* Demodex* differ with regard to their distribution on human skin [17], with* D. folliculorum* being more numerous, but* D. brevis* occupying a larger area on the body. In addition, different* D. brevis* to* D. folliculorum* ratios have been observed depending on the sex of the host: 1 : 10 in women and 1 : 4 in men [17].

*Demodex* mites are found in all humans living at a range of latitudes [17]. It is believed that transfer between people requires direct contact, through the use of common toiletries and towels, and through dust. Their colonization of the skin can occur as soon as in childhood or early adulthood; however, they are not found in the skin of newborn babies [21].

The mere presence of* Demodex* mites on the face does not lead to the development of disease symptoms for the majority of people [22]. However, although it was first thought that the* Demodex* mites live in symbiosis with man [17], many reports have since revealed their pathogenic role. Their presence is correlated with both eyes and skin diseases, and they have been identified in patients with rosacea [23, 24]. It is believed that the mites may also participate in the development and intensification of other types of dermatitis, and recently interest has grown in this regard [25, 26]. Among eye diseases, a positive correlation was mostly observed between the presence of* Demodex* and inflammation of the eyelid margins [17, 24, 25, 27, 28]. Therefore, a knowledge of the potential of* Demodex* as an etiological agent is not only important for the sake of research, but it can also contribute to more effective treatment of certain diseases of the eyes and skin.

The aim of the present study was to identify any relationship between the incidence of* Demodex* mites in eyelash hair follicles in patients using soft contact lenses and the abandonment of these lenses by their users. In all cases, the lenses had been worn for a minimum period of six months, with no related symptoms of discomfort.

## 2. Materials and Methods

A group of 62 individuals was studied: 28 of whom reported discomfort and had abandoned the use of contact lenses and 34 did not report discomfort and were still benefiting from the use of contact lenses. This group included 43 women and 19 men. All participants had worn soft contact lenses for a minimum of six months and maximum of* c.a.* 10 years, with no related symptoms of discomfort. After this period, the patients started to feel discomfort and intolerance to soft contact lenses and finally abandoned them. As the presence of* Demodex* spp. may be associated with chalazion and blepharitis, all patients underwent slit-lamp examination and the appearance of their eyelids was assessed. Patients with acute eyelid and/or ocular surface infection or inflammation were excluded. The average age of respondents was 29.18 years (women, 28.81 years; men, 30.00 years).

A minimum of ten lashes in total were taken from each subject, with at least five lashes removed from each (lower and upper) eyelid. The removed lashes were examined under light microscopy, applying standard parasitological methods if* Demodicosis* is suspected. All results were recorded with the camera built into the device. A positive result was assumed if at least one adult stage, larva, protonymph, or nymph of* D. folliculorum* or* D. brevis* was present (Figures 1–6). All patients gave their signed, written consent for the collection of the lashes for the diagnostics procedure and further tests.

### 2.1. Clinical Test

All subjects underwent a slit-lamp examination to determine their Noninvasive Break Up Time (NIUBUT) during contact lens use. In the patients with* Demodex* spp. present, microscopic photographs of the parasite were taken.

### 2.2. Statistical Analysis

To examine the relationship between the presence of* Demodex* and intolerance to contact lenses, the Chi-square independence test was applied with a fourfold table. As the Chi-square test does not provide information about the strength of dependence, the Czuprow coefficient, with a value in the range of 〈0; 1〉, was also calculated. The level of significance for all tests was adopted as *α* = 0.05.

## 3. Results

All individuals were tested for the presence of* Demodex* and intolerance to contact lenses. The following four groups were distinguished on the following basis: Group 1, patients with* Demodex* and tolerance to contact lenses; Group 2, patients with* Demodex* and intolerance to contact lenses; Group 3, patients without* Demodex* and with intolerance to contact lenses; and Group 4, patients without* Demodex* and with intolerance to contact lenses. The sizes of the individual groups broken down by sex, average age, and share of the total population are shown in Table 1.

It was found that 92.86% of subjects with intolerance to contact lenses were positive for the presence of* Demodex* (Figure 9). In addition, only 5.88% of patients with* Demodex* present reported no symptoms of discomfort (Figure 10).

With regard to the NIBUT test, the first breakup in patients with intolerance to contact lenses was 10.39 s and the average breakup 13.35 s, while the corresponding values for patients tolerating contact lenses were 13.15 s for the first breakup and 15.45 s for average breakup. Of the patients who were intolerant to contact lenses, 41.93% of the total group (26 patients) demonstrated* Demodex* infection (Figure 11), whereas only 3.22% (2 patients) were free of* Demodex*.

Contact lens intolerance was found in 28 of the 62 examined patients, and* Demodex* was also found to be present in 28 (Table 2). Twenty-six individuals (42%) were found to have both contact lens intolerance and* Demodex*, whereas only 2 individuals demonstrated intolerance and were free of* Demodex*. The results of the Chi-square test indicate that a significant relationship exists between the occurrence of* Demodex* and intolerance to contact lenses.

As the Chi-square test does not provide information about the strength of dependencies, the Czuprow coefficient was calculated. The coefficient was found to be *T* = 0.87, which indicates a strong dependency and confirms the presence of a positive correlation between* Demodex* mite infection and contact lens intolerance.

## 4. Discussion

Baima and Sticherling [29] suggest that lesions which occur in the course of eyelid* Demodicosis* are the consequence of obstruction caused by blockage of the hair follicles and outlet tubes of the sebaceous glands by mites, excessive keratinization and hyperplasia of the epithelium, mechanical transfer of bacteria and fungi, the inflammatory response of the host to the presence of the chitin of the parasite, as a foreign body, and stimulation of the humoral and cellular response of the host under the influence of the mites and their metabolites. Akilov and Mumcuoglu [30] suggest that* Demodex* can cause local suppression of the host immune system, which allows them to develop and survive in the skin of the patient. The presence of* Demodex* can have a significant influence on the accumulation of antigens and biological material, such as moult, eggs, and fragments of decayed organisms. This collection may predispose the patient to complications associated with the use of contact lenses, increase the likelihood of mechanical irritation of the eye and its associated perceived discomfort, and ultimately lead to the patient not using the lenses.

Acute symptoms of discomfort and signs of friction on the surface of the eyeball can be eliminated or minimized by choosing lenses with a low coefficient of friction, using an optimal tear film and lipid layer, and preventing infection of the eyelid margins [14]. Young et al. matched one-day contact lenses for patients who had stopped using them due to perceived discomfort and obtained a high rate of success in a short period of time. This suggests that the technological improvements that have occurred in the period between the lenses being abandoned and fitted again may prove beneficial, as well as reducing the time of wearing them [31].

**Figure 7:**
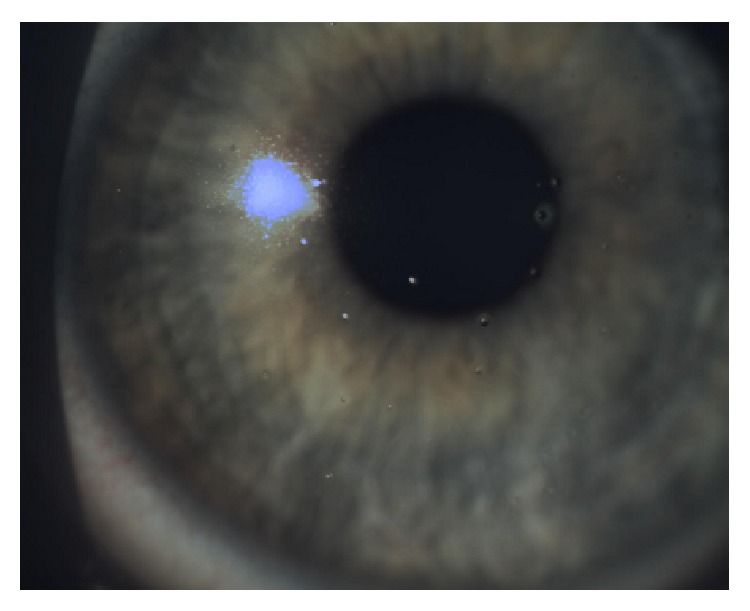
Protein deposits.

**Figure 8:**
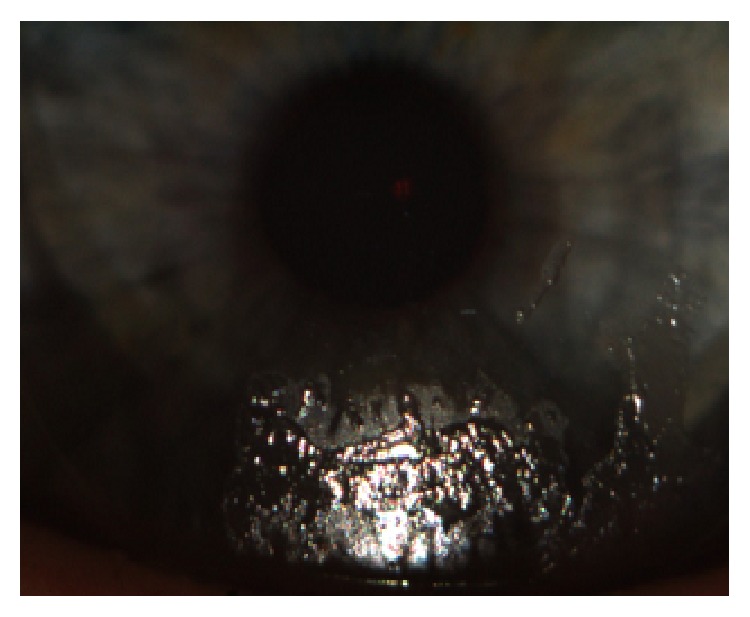
Protein-lipid deposits.

**Figure 9:**
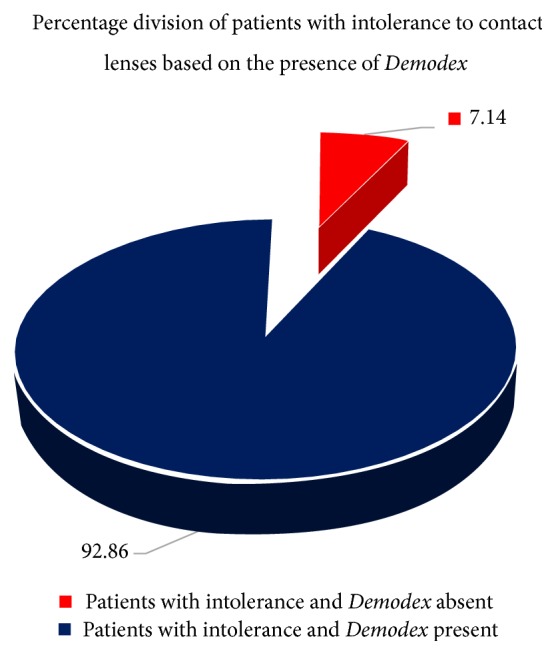
The percentages of patients with intolerance to contact lenses according to the presence of* Demodex*.

**Figure 10:**
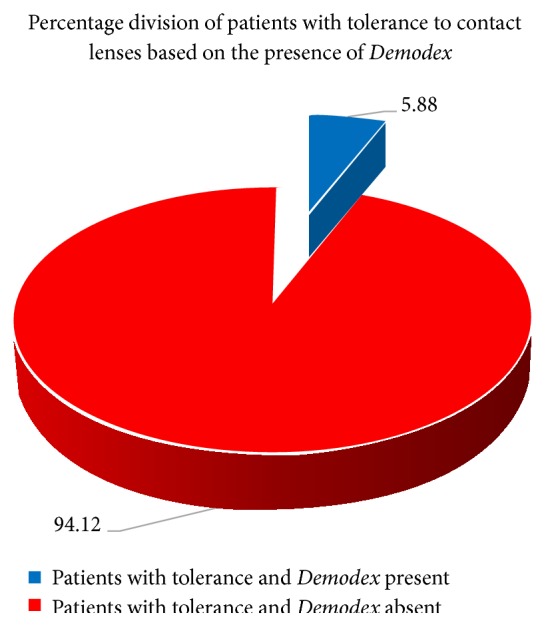
The percentages of patients with intolerance to contact lenses according to the presence of* Demodex*.

**Figure 11:**
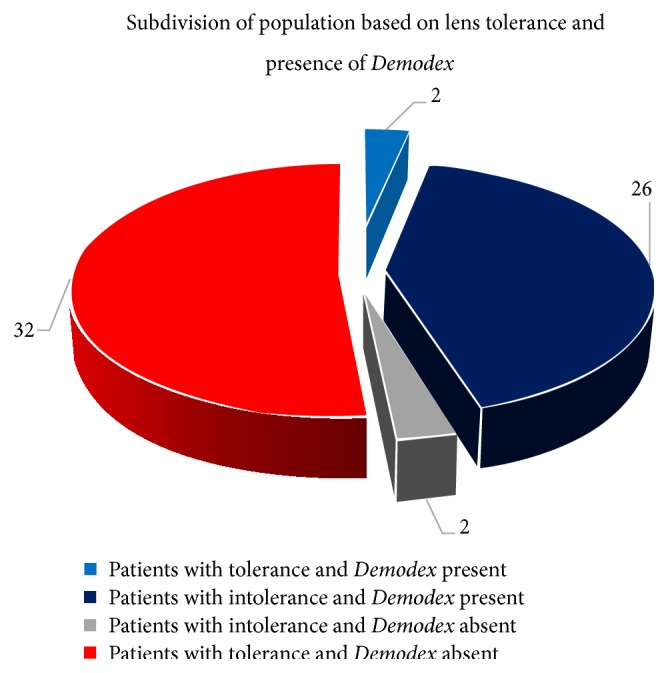
Population of tested subjects with division into subgroups.

Our results highlight an important relationship between the presence of* Demodex* in the hair follicles of eyelashes and intolerance to contact lenses. Apart from the factors mentioned above, Baima and Sticherling [29] suggest that* Demodex* spp. block the outlets of Meibomian and the Zeiss glands, leading to stagnation of holocrine gland secretion and incorrect tear film composition. A chronic inflammatory reaction by the host to the presence of chitin and parasite metabolites leads to a significant increase in immune proteins in the tear film, which in turn leads to the rapid emergence of lipid and protein deposits on the surface of the contact lenses, which are hydrophobic areas (Figures 7 and 8). These changes cause excessive evaporation of the sublens tear film and lead to the symptoms of discomfort experienced by the patient.

The abandonment of contact lenses by the patients in our study cannot be attributed to incorrect lens geometry (e.g., the radius of curvature, thickness, diameter, and type of edge) or material (e.g., coefficient of friction or stiffness) or the composition of nursing fluids. All tested patients used the same model of lens and nursing fluids and followed the same procedure for a minimum period of six months, during which time no symptoms of discomfort were reported.

A number of recent reports describe the impact of environmental factors on the emergence of complications in contact lens users. Long periods of work in front of a computer monitor and the longer periods between blinking associated with them, which can be as much as five times longer, and the reduced stability of the sublens tear film have been identified as factors [32]. In addition, such factors as low humidity and high temperature have been associated with discomfort [33]. A study conducted in Japan [34] on a group of over 2,000 current and former contact lenses users found that length of time spent in front of the computer was the most commonly reported cause of discomfort. The subjects in the present study reported no recent changes in lifestyle, such as a significant extension of time spent at the computer, which could influence lens intolerance. This further confirms the role played by* Demodex* in the decision to abandon the use of soft contact lenses. Demodicosis is clearly a growing, and as yet underestimated, ophthalmological issue.

## 5. Conclusions

Our findings clearly indicate the existence of a relationship between* Demodex* infestation in the hair follicles of the eyelashes of contact lens users and their decision to abandon the use of soft contact lenses. Discomfort felt by the users should be an indication for parasitological examination to exclude the presence of Demodicosis as a potential cause.

## Figures and Tables

**Table 1:** The size of individual groups in the examined population.

	Group 1	Group 2	Group 3	Group 4
Population	2 (F: 2, M: 0)	26 (F: 17, M: 9)	2 (F: 2, M: 0)	32 (F: 22, M: 10)
Proportion of the total number of subjects	3.22%	41.93%	3.22%	51.61%
Average age in the groups	38.00	32.27	26.50	26.28

M: the number of males, F: the number of females.

**Table 2:** The relationship between the occurrence of *Demodex* and intolerance to contact lenses in the examined group of patients.

	*Demodex *		
	Present	Absent	∑
Intolerance to contact lenses			
Present	26	2	**28**
Absent	2	32	**34**
∑	**28**	**34**	**62**
